# Complete chloroplast genome sequence of *Artemisia fukudo* Makino (Asteraceae)

**DOI:** 10.1080/23802359.2016.1155426

**Published:** 2016-06-20

**Authors:** Yun Sun Lee, Jee Young Park, Jin-Kyung Kim, Hyun Oh Lee, Hyun-Seung Park, Sang-Choon Lee, Jung Hwa Kang, Taek Joo Lee, Sang Hyun Sung, Tae-Jin Yang

**Affiliations:** a Department of Plant Science, Plant Genomics and Breeding Institute, and Research Institute of Agriculture and Life Sciences, College of Agriculture and Life Sciences, Seoul National University, Seoul, Republic of Korea;; b Phyzen Genomics Institute, Seongnam, South Korea;; c Hantaek Botanical Garden, Yongin, Gyeonggi-Do, Republic of Korea;; d College of Pharmacy and Research Institute of Pharmaceutical Science, Seoul National University, Seoul, Republic of Korea;; e Crop Biotechnology Institute/GreenBio Science and Technology, Seoul National University, Pyeongchang, Korea

**Keywords:** *Artemisia fukudo*, chloroplast, genome sequence

## Abstract

In this study, a complete chloroplast genome sequence of *Artemisia fukudo* (Asteraceae family) was characterized by *de novo* assembly using whole genome sequence data. The chloroplast genome was 151,011 bp in length, comprising a large single-copy region of 82,751 bp, a small single copy region of 18,348 bp and a pair of inverted repeats of 24,956 bp. The genome contained 80 protein-coding genes, 4 rRNA genes and 30 tRNA genes. Phylogenetic tree revealed that *A. fukudo* was closely located in other *Artemisia* species, *Artemisia montana* and *Artemisia frigida*.

The *Artemisia* genus belongs to the Asteraceae family and is a native to Europe, North Africa, and Asia, including Korea (Abad et al. 2012). In Korea, about 20 *Artemisia* species were found and have been used as ingredients for food and as medicinal herbs for a long time (Kim et al. 2007). *A. fukudo* is mainly distributed along the shorelines of Jeju Island, South Korea, and have been used as a flavouring agent and cosmetic ingredient in Korea (Abad et al. 2012). Previous study reported that *A. fukudo* possessed essential oils composed of oxygenated monoterpene and sesquiterpene hydrocarbons with anti-inflammatory effect, and confirmed its potential herbal medicinal activity (Yoon et al. 2010). Lots of *Artemisia* species grow together in wild and there is some difficulty to authenticate each species because of their similar morphological traits. Practical barcode markers are necessary to authenticate related species from the processed products (Jung et al. 2014) because dried plant leaves of *Artemisia* species are used as crude drug. Although complete chloroplast genome sequences of two *Artemisia* species (JX293720 and KF887960) are known, there was no report for *A. fukudo*. We developed chloroplast genome sequence of *A. fukudo* to further apply developing authentication as well as in improving its quality as medicinal plant.

Chloroplast genome sequence of *A. fukudo* was generated using *de novo* assembly method for chloroplast genome reported by Kim et al (2015a and 2015b). *Artemisia fukudo* was collected from Jeungdo, Jeollanam-do, South Korea, and maintained in Hantaek Botanical Garden (http://www.hantaek.co.kr/). We isolated DNA from leaf tissues and performed whole genome shotgun sequencing using an Illumina Miseq platform (Illumina, San Diego, CA). High-quality paired end reads of 1.2 Gb were assembled using CLC genome assembler 4.6 (CLC Inc., Aarhus, Denmark) and contigs representing chloroplast sequence were retrieved, ordered, and combined into a single sequence, by the guidance of chloroplast genome of *A. frigida* (JX293720) (Liu et al. 2013).

Complete chloroplast genome of *A. fukudo* (Accession number: KU360270) was 151,011 bp in length, consisted of four distinct parts including a large single copy region of 82,751 bp, a small single copy region of 18,348 bp, and a pair of inverted repeats of 24,956 bp. In genome, a total 114 coding regions, which were comprised 80 protein-coding regions, 4 rRNA genes, and 30 tRNA genes, were predicted through annotation using DOGMA (http://dogma.ccbb.utexas.edu) and BLAST searches.

Phylogenetic relationship was analyzed with the chloroplast genome of *A. fukudo* along with 11 species belonging to the Asteraceae family. A total of 74 coding sequences were used for the analysis, and phylogenetic tree was constructed using neighbour-joining method in the MEGA 6.0 (Tamura et al. 2013). Phylogenetic tree revealed that *A. fukudo* was grouped with two *Artemisia* (*A. montana* and *A. frigida*), as expected. However, *Guizotia abyssinica* and *Helianthus annuus,* which belong to the subfamily Asteroideae, were placed with *Lactuca sativa* (Subfamily *Cichorioideae*) and two *Cynara* species (Subfamily *Carduoideae*), suggesting that there is still a contradiction in taxonomical classification of Asteraceae family (Xia et al. 2015) (Figure 1).

**Figure 1.**
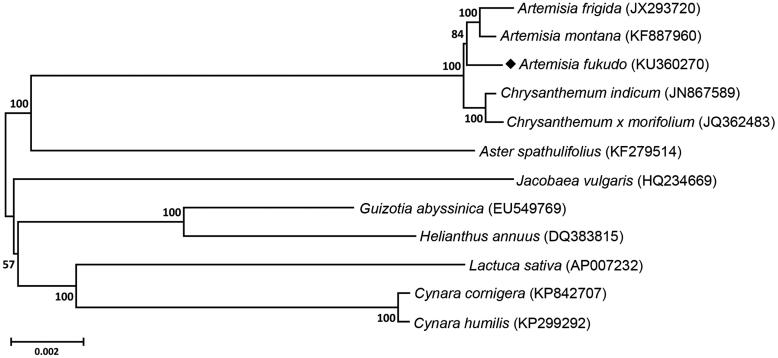
Phylogenetic tree revealing the relationship of *A. fukudo* with 11 species belonging to the Asteraceae family. This tree were constructed with those complete chloroplast genome sequences using neighbour-joining method with 1000 bootstrap values in the MEGA 6, and numbers above each node indicate the bootstrap values.
